# Change in Quality of Life after Medical and Surgical Treatment of Graves’ Ophthalmopathy

**DOI:** 10.4103/0974-9233.75884

**Published:** 2011

**Authors:** Mohsen Bahmani Kashkouli, Iraj Heidari, Farzad Pakdel, Sara Jam, Yasamin Honarbakhsh, Bahareh Mirarmandehi

**Affiliations:** Department of Eye, Eye Research Center, Rassoul Akram Hospital, Iran University of Medical Sciences, Tehran, Iran; 1 Institute of Endocrinology, Iran University of Medical Sciences, Tehran, Iran

**Keywords:** Eyelid Retraction, Graves’ Ophthalmopathy, Proptosis, Quality of Life

## Abstract

**Purpose:**

To assess the changes in quality of life (QOL) of patients after treatment of their Graves’ ophthalmopathy (GO).

**Materials and Methods:**

In this prospective, cross-sectional study, the GO-QOL questionnaire was completed by 67 subjects before and at least 6 months after steroid treatment (61 subjects, group 1) or optic neuropathy orbital decompression (6 subjects, group 2). Visual, psychosocial, education and counseling scores (higher score = better health), GO severity and clinical activity scores and minimal clinically important difference (MCID) were recorded and analyzed for correlation and statistical significance. A *P*-value <0.05 was considered statistically significant.

**Results:**

The mean age of patient enrolled in the study was 38.3 years, with 43 females (64.2%). The mean duration of thyroid dysfunction and GO were 40.1 and 26.5 months, respectively. Two treatment groups were similar for all the variables (0.06 < *P* < 0.9), except for higher mean age in the orbital decompression group (45.2 versus 37.7 years) (*P* = 0.03). Mean severity, activity, visual function and psychosocial function scores significantly improved in group 1 (steroid group) (*P* < 0.05, all cases). A significant improvement in clinical activity score and psychosocial scores occurred in group 2 (decompression group) (*P* < 0.05). MCID was achieved in two-thirds of the patients, with no significant difference between groups (*P* > 0.05). There was no significant effect of duration of thyroid disease and GO and severity and activity of GO on QOL scores either before or after treatment (*P* > 0.05, all cases).

**Conclusion:**

Steroid treatment and orbital decompression significantly improve the QOL in GO. Duration, severity and activity of GO did not have a significant impact on the QOL.

## INTRODUCTION

Graves’ ophthalmopathy (GO), associated with Graves’ Disease (GD), is an incapacitating eye disease causing disfiguring proptosis, pain, redness and swelling of the eyelids, grittiness of the eyes, diplopia and, at times, a chronic debilitating infiltrative eye disease that, in some situations, can be associated with blindness.^1^–^6^

The perceptions of patients of how they feel and how they are able to function in daily life should be included in the evaluation and monitoring of the effects of disease and treatment, which are influenced by a patient’s experience, beliefs and expectations.^7^–^10^

The outcomes of GO and its treatments are mostly assessed by biological measures such as activity and severity scores, which do not correlate well with the patient’s subjective impression.^11^ GO leads to a worse quality of life (QOL) score than many chronic disorders such as diabetes mellitus.^12^–^14^

A disease-specific QOL questionnaire for GO (GO-QOL) was developed consisting of visual (eight questions) and psychosocial (eight questions) subscales.^1^ It was found to be a valid and reliable instrument available in six languages and could be used as a separate outcome measure in clinical studies.^15^ A modified GO-QOL was used by Park *et al*.,^2^ who found it to be a simple and practical tool that could be used easily in a clinic to determine the QOL in subjects with GO.

Although there are few scales to measure the severity and activity of GO, the NOSPECS severity score^16^ and Mourits’ clinical activity score^17^ are widely used in practice. Minimal clinically important difference (MCID) has been defined as “the smallest difference in score on the domain of interest which patients perceive as beneficial and which would mandate, in the absence of troublesome side effects and costs, a change in the patient’s management.”^18^ Terwee *et al*.^19^ recommended a change of ≥6 points after minor treatment and ≥10 points after orbital decompression and major treatment as MCID in patients with GO.

The aims of this study were to assess the change in QOL in patients with GO 6 months after treatment (steroid therapy and optic neuropathy orbital decompression) and to assess the effect of severity and activity scores on QOL.

## MATERIALS AND METHODS

In a cross-sectional study, candidates with GO were screened from May 2005 to May 2006. Exclusion criteria were subjects with less than 18 years of age, absence of clinical and biochemical euthyroid state (achieved by medications, radioactive iodine or surgery) at the time of questionnaire completion, presence of other chronic disorders such as diabetes mellitus and incomplete follow-up. Each patient completed a questionnaire before and at least 6 months after the medical or surgical treatments.

The modified GO-QOL questionnaire by Park *et al*.^2^ [Table 1] was translated in local language (Farsi). We found it easier to use just one question regarding the education and counseling [Table 1]. As a measure of the reliability, the internal consistency based on correlations of items within subscales, was assessed in a pilot study of 16 patients (Cronbach’s alfa = 0.888).

**Table 1:** Frequencies of responses from questions on visual (nine), psychosocial (eight) and education (one) in 67 patients with Graves’ ophthalmopathy before and at least 6 months after steroid treatment or optic neuropathy orbital decompression

Visual		Missing response (%)		No, not at all limited (%)		Yes, a little limited (%)		Yes, severely limited (%)	
		After	Before	After	Before	After	Before	After	Before
1	Driving	53.7*	53.7*	35.8	25.4	10.4	11.9	0	9
2	At work (usual job)	3	4.5	79.1	49.3	16.4	26.9	1.5	19.4
3	Performing domestic duties (cooking, etc.)	0	9	73.1	55.2	22.4	29.9	4.5	6
4	Moving around the house	7.5	0	68.7	58.2	23.9	29.9	0	11.9
5	Walking outdoors	3	0	70.1	50.7	26.9	32.8	0	16.4
6	Reading	1.5	1.5	68.7	38.8	25.4	40.3	4.5	19.4
7	Watching TV	1.5	0	35.8	49.3	59.7	38.8	3	11.9
8	Hobby or pastime	1.5	3	73.1	55.2	22.4	34.3	3	7.5
9	During the course of your illness, did you feel hindered from doing something that you wanted to do because of your thyroid eye disease?	0	0	67.2	43.3	19.4	25.4	13.4	31.3

**Psychosocial**		**Missing response (%)**		**No, not at all limited (%)**		**Yes, a little limited (%)**		**Yes, severely limited (%)**	
		**After**	**Before**	**After**	**Before**	**After**	**Before**	**After**	**Before**

10	Do you feel that your appearance has changed because of your thyroid eye disease?	0	0	46.3	7.5	31.3	34.3	22.4	58.2
11	Do you feel that you are stared at in the streets because of your thyroid eye disease?	0	0	52.2	23.9	37.3	34.3	10.4	41.8
12	Do you feel that people read unpleasantly because of your thyroid eye disease?	1.5	1.5	76.1	53.7	19.4	34.3	3	10.4
13	Do you feel that your thyroid eye disease has an influence on your self-confidence?	0	3	67.2	32.8	28.4	44.8	4.5	19.4
14	Do you feel that your thyroid eye disease has an influence on making friends?	0	1.5	83.6	47.8	14.9	29.9	1.5	20.9
15	Do you feel socially isolated because of your thyroid eye disease?	0	0	82.1	59.7	11.9	26.9	6	13.4
16	Do you feel that you appear less often on photos than before you had thyroid eye disease?	0	1.5	50.7	25.4	38.8	34.3	10.4	38.8
17	Do you try to mask changes in your appearance caused by thyroid eye disease?	0	0	73.1	41.8	10.4	25.4	16.4	32.8
Do you feel education and counseling concerning thyroid eye disease was adequate?									
18	Do you feel patient education and counseling concerning thyroid eye disease was adequate?	0	7.5	20.9	29.9	23.9	38.8	55.2	23.9

* No driving license

Scores of the questions were summed and transformed to a 0–100 scale, with 0 indicating the worst and 100 indicating the best state. Data were obtained on the duration of thyroid dysfunction and GO, current GO severity score^16^ and activity score.^17^

Total eye score was used to assess the severity of GO, which was calculated by multiplying each class of the NOSPECS system (except class 0) to its grade of severity (0–3), yielding a maximum total score of 63 and a minimum total score of 0 (the higher the number the worse the severity). Clinical activity score is based on signs and symptoms of inflammation (range: 0–10, the higher the number the greater the activity).

MCID was considered ≥6 points for steroid treatment and ≥10 points for orbital decompression in this study.

This study received ethics committee approval from the Iran University Eye Research Center. Data were entered with SPSS, version 15 (SPSS Inc., Chicago, IL, USA). Statistical analysis was performed with the Kolmogorov-Smirnov test (analyzing the pattern of normal distribution), the paired t-test (analyzing the QOL scores before and after treatment), Mann–Whitney test (analyzing the scores in different gender and treatment groups), linear regression test and Spearman’s correlation test (analyzing the correlation between the severity, activity and QOL scores) and Chi-square test (analyzing the MCID in different treatment groups and comparing the education and counseling before and after treatment). *P* <0.05 was considered statistically significant.

## RESULTS

The study cohort comprised of 61 subjects with steroid treatment (group 1) and six subjects with optic neuropathy orbital decompression (group 2). There were 43 females (64.2%). Mean age of the patients was 38.3 ± 13.4 years (range, 18–73 years). The mean duration of thyroid dysfunction was 40.1 ± 44.8 months (range, 2–240 months) and the GO was 26.5 ± 38.2 months (range, 2–240 months) at the time of taking the test. No gender difference was found for the variables (0.1 < *P* < 0.8) [Table 2]. Two groups were similar for all the variables (0.06 < *P* < 0.9), except for higher mean age in group 2 (45.2 years versus 37.7 years) (*P* = 0.03).

**Table 2:** Comparison of the mean total eye score (the higher the worse), mean activity score (the higher the worse), mean quality of life visual and psychosocial hundred scores (the higher the better) and positive minimal clinical important difference (MCID) in 67 patients with Graves’ ophthalmopathy before and at least 6 months after steroid treatment or optic neuropathy orbital decompression

		Steroid (61 patients)	Orbital decompression (6 patients)	*P*-value	All (67 patients)
Mean total eye score	Before	16.3	17.7	0.7§	16.4
	After	7.5	9.6	**0.2**§	7.7
	*P*-value*	0.000	0.2	-	0.000
Mean activity score	Before	4.7	5.6	0.1§	4.7
	After	0.8	1.3	0.2§	0.8
	*P*-value*	0.000	**0.001**	-	0.000
Mean visual hundred score	Before	62.3	43.5	0.1§	60.6
	After	82.4	78.1	0.5§	82
	Mean change	20.1	34.6	-	21.4
	*P*-value*	0.000	**0.06**	-	0.000
Mean psychosocial hundred score	Before	54.8	43.1	0.3§	53.7
	After	79.2	79.1	0.6§	79.2
	Mean change	24.4	36	-	25.5
	*P*-value*	0.000	**0.04**	-	0.000
+MCID (%)	Visual	70.4% (43/61)	83.3% (5/6)	0.4#	71.6% (48/67)
	Psychosocial	77% (47/61)	66.6% (4/6)	0.4#	76.1% (51/67)
	*P*-value#	0.1	**0.6**	-	0.6

* paired t-test

# Chi-square test

§ Mann–Whitney test

*P* <0.05 is statistically significant

The most frequently reported severe limitation was restriction in performing the activities that the patient was interested in (visual function scores) and the change in facial appearance (psychosocial function score) [Table 1].

**Figure 1:**
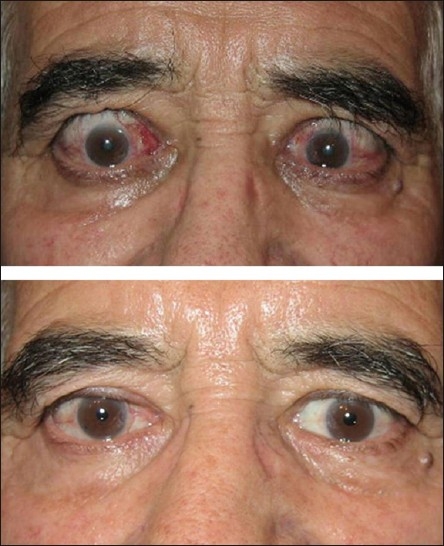
A 62-year-old man with active Graves’ ophthalmopathy before (top) and after (bottom) steroid treatment and bilateral upper eyelid botulinum toxin injection

**Figure 2:**
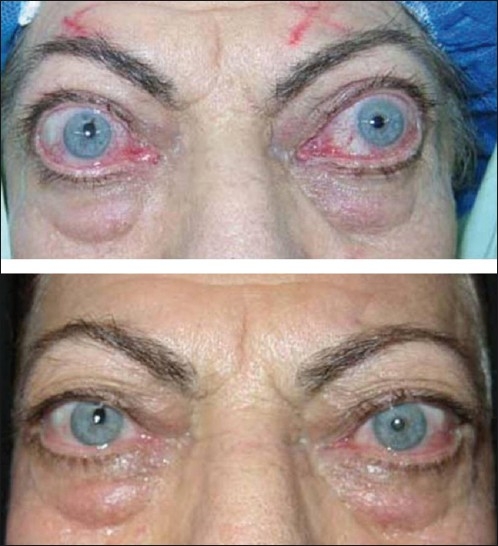
A 58-year-old woman with bilateral dysthyroid optic neuropathy before (top) and after (bottom) bilateral orbital decompression

**Figure 3:**
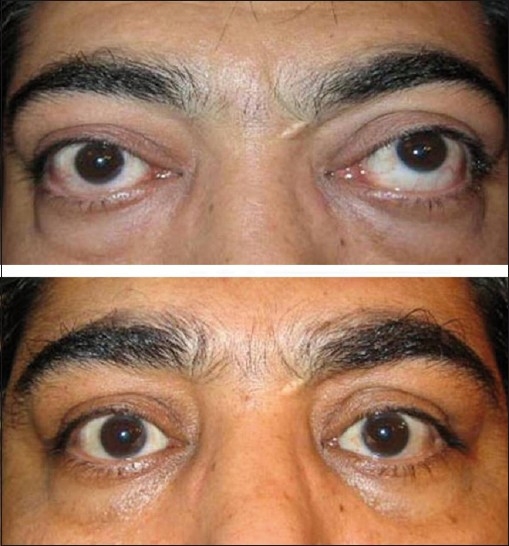
A 32-year-old man with left dysthyroid optic neuropathy before (top) and after (bottom) left orbital decompression and bilateral lower eyelid retractor recession

Mean severity, activity, visual function and psychosocial function significantly improved after treatment in group 1 [Table 2, *P* < 0.05, all cases]. However, group 2 showed a significant improvement in the clinical activity score and psychosocial score but not in severity and visual function scores after orbital decompression [Table 2, *P* < 0.05, all cases]. More than two-thirds of the subjects achieved MCID [Figures 1 – 3] after treatment, which was not statistically different in the two groups [Table 2].

Although higher education and counseling scores were observed after treatment, the change did not reach significance (0.07 < *P* < 0.3) [Table 3]. A significantly positive correlation was found between visual and psychosocial function scores both before (r = +0.51, *P* = 0.000) and after (r = +0.62, *P* = 0.000) treatment.

**Table 3:** Comparison of the percentage of responses to education and counseling scores in 67 patients with Graves’ ophthalmopathy before and at least 6 months after steroid treatment (56 valid responses, five missing responses) and optic neuropathy orbital decompression (six valid responses)

	Good education and counseling		Little or no education and counseling		*P*-value (Chi-square test)
	Before	After	Before	After	
Steroid	26.8% (15/56)	58.9% (33/56)	73.2% (41/56)	41.1% (23/56)	0.1
Orbital decompression	16.7% (1/6)	33.3% (2/6)	83.3% (5/6)	66.7% (4/6)	0.3
All patients	25.8% (16/62)	56.5% (35/62)	74.2% (46/62)	43.5% (27/62)	0.07

There was a significantly (*P* = 0.02) worse QOL score for psychosocial (53.7) than visual function (60.6) scores before treatment. However, there was a still worse score for psychosocial (79.2) than visual (82) function after treatment, the difference being not significant (*P* = 0.2). There was no significant effect of duration of thyroid disease and GO and severity and activity of GO on QOL scores either before or after treatment (0.07 < *P* < 0.9).

## DISCUSSION

GO dramatically decreases the patient’s QOL and may also significantly alter their personal behavior.^1^,^2^,^5^,^9^ It has been reported that after treatment, 61% of the patients believed that the appearance of their eyes had not returned to baseline status and 51% thought that their eyes continued to be abnormal in appearance.^20^

The most common complaint on severely impaired visual item reported by our patients before treatment was “feeling hindered from doing something” (31%), which is similar to the outcomes reported by Park 2(34%) and Terwee^15^(35%).

On the psychosocial subscale, our patients felt mostly impaired by their changed appearance (58.2%). This item was also the most limited psychosocial item in other studies.^1^,^15^ Closely similar to others,^1^,^2^,^15^ impaired self-confidence was reported in 64% of our patients [Table 1].

While few studies reported a low correlation between QOL scores and severity or activity of GO, 1,12,19,21 Park^2^ found that a patient with more severe GO had a significantly worse QOL score. Studies on chronic illnesses also found a weak correlation between clinical measures and the QOL.^22^,^23^ We found no correlation between QOL scores and activity and/or severity scores before and after treatment [Table 2]. QOL is a subjective measure of patient’s experiences that is distinct from clinical objective measures. Different range of disease severity and activity in different studies may lead to different correlations.

We did not find a significantly different QOL score in different ages whereas, Park *et al*.^2^ reported that older patients with GO were more concerned about visual but not psychosocial limitation. Relatively younger ages (mean = 38 years) in our study may account for having the same concerns for visual and psychosocial limitations.

Change was statistically significant for both visual and psychosocial scores in the steroid group (*P* = 0.000). In the orbital decompression group, however, change in psychosocial score was statistically significant (*P* = 0.04), but change in visual score was just marginally insignificant (*P* = 0.06). A small number of subjects in the orbital decompression group may account for this result. Terwee *et al*.^19^ reported a significant visual improvement but no significant psychosocial change after orbital decompression.

Similar to Park’s report,^2^ our subjects generally showed better visual than psychosocial score before and after treatment, whereas Terwee *et al*.^19^ reported a better psychosocial score before treatment and almost equal scores for visual and psychosocial after treatment [Table 4]. Patient’s perspectives and the characteristics of the patients and their environment, such as expectations, coping ability, motivation, social support and physician-patient relationships may account for this difference.^1^,^5^,^9^,^12^,^21^

**Table 4:** Comparison of different studies on quality of life (by GO-QOL questionnaire) in patients with Graves’ ophthalmopathy

	Present study (before treatment)	Present study (after treatment)	Terwee *et al*.^1^	Terwee *et al*.^19^,^21^		Park *et al*.^2^
				Before treatment	After treatment	
No.	67		70	164		128
Mean age (SD)	38 (13.4)		53.3 (13.1)	50 (12)		48.5 (NA)
Gender						
Female	43 (64%)		50 (71%)	133 (81%)		106 (82%)
Male	24 (26%)		20 (29%)	31 (19%)		22 (18%)
Mean duration of Ophthalmopathy (months)	26.5 (2–240)		12 (1–206)	39 (4–571)		NA
Mean duration of thyroid dysfunction (months)	40.1 (44.8)		18 (0–456)	NA		NA
Mean total eye score (TES)	16.4	7.7	9 (2–28)	NA	NA	NA
Mean activity score (SD)	4.7 (1.46)	0.8 (0.9)	2.6 (1.3)	NA	NA	NA
Visual hundred score (SD)	60.6 (23.9)	82 (15.3)	54.7 (22.8)	54.7 (22.8)	78.2 (23.7)	59.0 (28.0)
Psychosocial hundred score (SD)	53.7 (25.4)	79.2 (23.5)	60.1 (24.8)	60.1 (24.6)	77 (22.4)	54.5 (28.4)

* NA: Not available

MCID has been defined as “the smallest difference in score on the domain of interest which patients perceive as beneficial and which would mandate, in the absence of troublesome side effects and costs, a change in the patient’s management.”^18^ Terwee *et al*.^19^ recommended a change of ≥6 points after minor treatment and ≥10 points after orbital decompression and major treatment as MCID in patients with GO. MCID was observed in almost 2/3 to 3/4 of the subjects in either group [Table 2]. The difference between different treatments and different subscales of QOL was not statistically significant (0.1 < *P* < 0.6).

Similar to Park’s study, 2we found that 74.2% of the GO patients reported a little or no education and counseling before treatment [Table 3]. Patients in the decompression group reported worse education and counseling both before and after treatment [Table 3]. Patients on steroid treatment are being gradually educated and counseled in parallel to their medical treatment effect, whereas orbital decompression for optic neuropathy is a relatively urgent and invasive treatment that does not allow the patient to be well informed and counseled about GO. This might the reason as to why there was better education and counseling score after steroid treatment than after orbital decompression, even though neither was statistically significant [Table 3]. It seems to be necessary to make more efforts on education in GO, especially when surgery is warranted.

In conclusion, GO profoundly affects the QOL. A worse psychosocial score than the visual function score was observed. Significant improvement of QOL scores were observed after steroid and orbital decompression. Severity and activity of GO did not significantly affect the QOL. More effort on education and counseling is recommended.
